# Response to pembrolizumab in a heavily treated patient with metastatic ovarian carcinosarcoma

**DOI:** 10.1186/s40661-018-0063-3

**Published:** 2018-08-18

**Authors:** Graziela Zibetti Dal Molin, Carina Meira Abrahão, Robert L. Coleman, Fernando Cotait Maluf

**Affiliations:** 1 Department of Gynecologic Oncology and Reproductive Medicine, The University of Texas MD Anderson Cancer Center, Houston, TX USA; 2 Hospital BP Mirante, São Paulo, Brazil; 3 Department of Gynecologic Oncology and Reproductive Medicine, The University of Texas MD Anderson Cancer Center, Houston, TX USA; 4 Hospital BP Mirante, Martiniano de Carvalho Street, 965, São Paulo, 01323-90 Brazil

**Keywords:** Immunotherapy, Pembrolizumab, Immune checkpoint inhibitors, Ovarian carcinosarcoma, Anti-PD1 antibody

## Abstract

**Background:**

Ovarian carcinosarcoma is a rare malignancy associated with a high rate of cancer-related mortality even at early stages. Guidelines for systemic treatment have been difficult to establish because the disease is commonly excluded from prospective clinical trials. Ovarian carcinosarcoma is usually managed as high-grade epithelial ovarian cancer despite major histologic differences. Owing to the rarity and poor prognosis of ovarian carcinosarcoma, salvage treatments and their efficacy have been poorly described.

**Case presentation:**

A patient heavily treated for ovarian carcinosarcoma showed an objective response to an immune checkpoint inhibitor, pembrolizumab. Pembrolizumab in this patient appeared to provide tumor control in multifocal metastatic sites.

**Conclusions:**

Pembrolizumab should be evaluated in prospective trials for the treatment of ovarian carcinosarcoma and further work is needed to identify patients most likely to respond to this type of intervention.

## Background

Ovarian carcinosarcoma (OCS), also known as malignant mixed mesodermal tumor or malignant mixed müllerian tumor, is a rare and highly aggressive malignancy that contains both sarcomatous and carcinomatous elements [1]. OCS accounts for 1–4% of all primary ovarian carcinomas [2]. According to an analysis of the Surveillance, Epidemiology and End Results data, the rate of OCS is 0.19 per 100,000 women [3]. Despite the rarity of OCS, it is associated with a high rate of cancer-related mortality even at early stages [4]. OCS typically occurs in postmenopausal women at a median age of 65 years and is staged according to FIGO criteria for epithelial ovarian cancer (EOC) [5]. More than 70% of patients present with advanced stage (stage III-IV) disease at the time of diagnosis [4]. The most common symptoms resemble those observed with EOC, including pelvic and/or abdominal pain, early satiety, bloating, and abdominal distention [6]. OCS often presents as a large tumor with massive areas of hemorrhage and necrosis. The morphological features and biology of the tumor seem identical regardless of its site of origin in the female genital tract [7].

Several clinical prognostic factors associated with poor outcome have been described, including age, advanced stage at presentation, and suboptimal surgical resection [6]. Other reports indicate that the use of adjuvant chemotherapy may have a clinical benefit, although the limited number of patients in these retrospective studies does not allow definitive conclusions in this regard [8].

The epithelial component of OCS seems to drive the prognosis and survival characteristics and is usually the dominant histologic characteristic in metastatic sites. OCS has a pattern of spread similar to EOC, with early dissemination to the serosa and peritoneum of the pelvic and abdominal cavity [2]. Despite the lack of specific data, OCS is usually treated as a high-grade EOC despite major differences in histologic characteristics, molecular features, response to systemic therapy, and outcome [8]. Median overall survival was also shown to be significantly decreased in women with OCS (24 months) compared with those with EOC (41 months) [9]. Owing to the rarity of OCS and the poor prognosis, salvage treatments and their efficacy have been poorly described. We describe a patient heavily treated for OCS that had an objective response to an immune checkpoint inhibitor, pembrolizumab.

## Case presentation

A 62-year-old woman, who was negative for the BRCA1/2 germline mutation, presented to our institution with abdominal pain in October 2011. She was referred from another institution, where she had undergone a primary suboptimal cytoreduction. Pathologic analysis revealed a carcinosarcoma with a high-grade serous adenocarcinoma component associated with high-grade endometrial sarcoma in the right ovary and fallopian tube, with angiolymphatic embolization. There were also peritoneal implants in the upper abdomen and pelvis. The uterus, left fallopian tube and ovary, and lymph nodes had no evidence of disease; however, peritoneal cytologic analysis was positive for malignancy. Immunohistochemistry of the right ovary demonstrated that the tumor was positive for estrogen and progesterone receptor but HER2/neu-negative.

From April through June 2012, the patient received adjuvant carboplatin (AUC 6) and paclitaxel (175 mg/m^2^) every 3 weeks for four cycles. In July 2012, she underwent an interval cytoreduction (optimal) followed by treatment with intraperitoneal cisplatin (75 mg/m^2^) and intravenous (135 mg/m^2^) and intraperitoneal paclitaxel (60 mg/m^2^) for six additional cycles. After the end of chemotherapy, she received tamoxifen as maintenance treatment for 4 months. She was observed off treatment for 6 months. In May 2013, retroperitoneal adenopathy was discovered following a serial rise in CA125, which prompted radiographic assessment. She received carboplatin (AUC 5), pegylated liposomal doxorubicin (30 mg/m^2^), and bevacizumab (10 mg/kg) for four cycles and had a partial response. Paclitaxel was not used again owing to residual neuropathy. In light of localized recurrence and response, she underwent a tertiary debulking procedure with complete macroscopic gross resection. Unfortunately, 6 months later a positron emission tomography/computed tomography study (PET-CT) revealed disease progression in a right retropectoral lymph node, as well as in paraesophageal lymph nodes. She then received cisplatin (35 mg/m^2^), gemcitabine (800 mg/m^2^), and bevacizumab (10 mg/kg), but had disease progression.

**Fig. 1:**
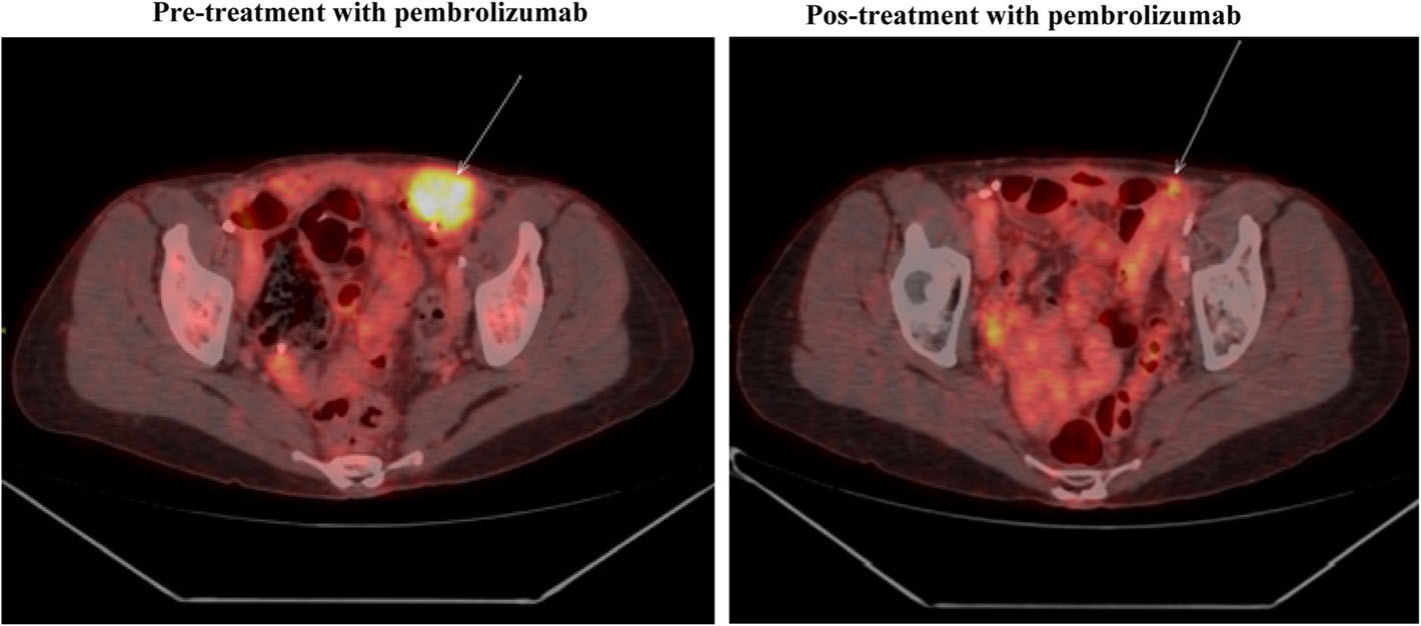
Objective partial response in the left external iliac lymph nodes. (Left) Before treatment with pembrolizumab. (Right) After four cycles of pembrolizumab

**Fig. 2:**
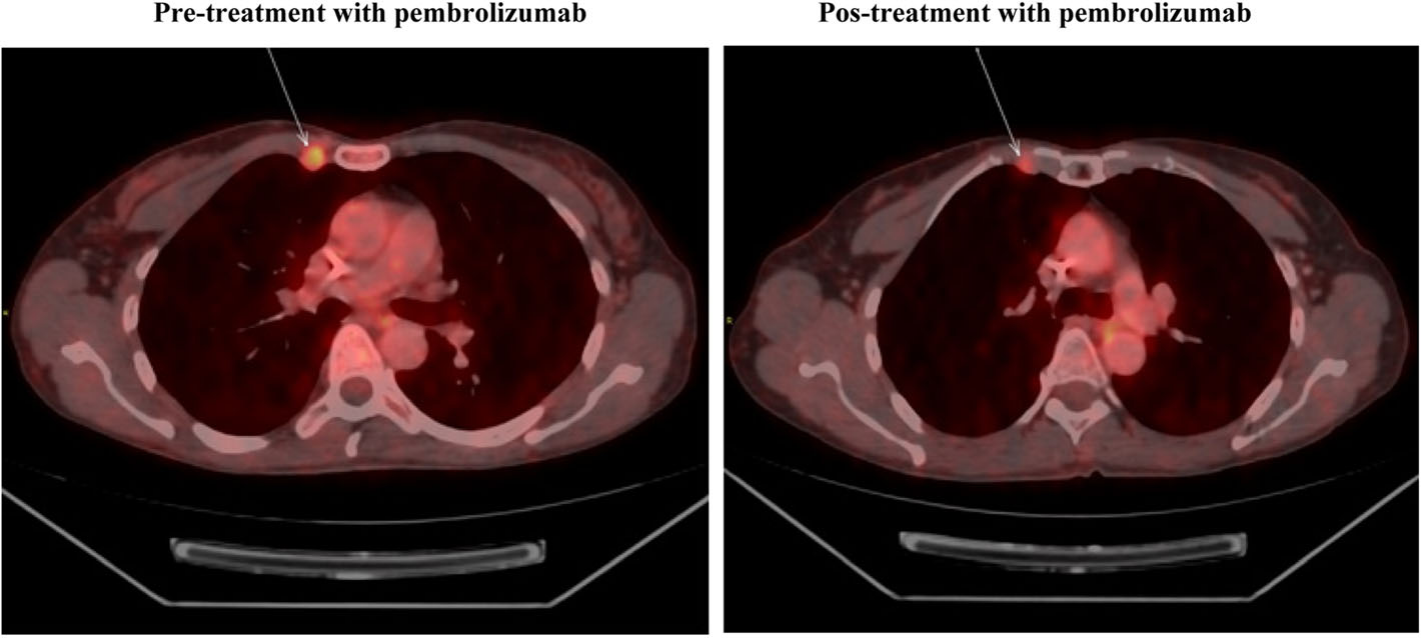
Objective partial response in the right-posterior pectoral lymph node conglomerate. (Left) Before treatment with pembrolizumab. (Right) After four cycles of pembrolizumab

From April 2014 through December 2015, the patient received various treatments for platinum-resistant disease: pemetrexed, nab-paclitaxel, megestrol, capecitabine, and vinorelbine. The metastatic sites were predominantly lymphatic and peritoneal. Despite the extensive pretreatment, she remained largely asymptomatic and had a good performance status. However, owing to disease progression, in January 2016 she initiated treatment with pembrolizumab (200 mg every 3 weeks). Unfortunately, there was not enough material to test the status of microsatellite instability, mutational load, or PD-L1 expression in the tumor. Also, the tumor was not analyzed for druggable mutations due to lack of insurance coverage. After the third cycle, she developed thyroiditis grade 1 but no other adverse events. After the fourth cycle, PET/CT showed objective partial response in the left external iliac lymph nodes (Fig. 1), as well as in the right-posterior pectoral lymph node conglomerate (Fig. 2). Another major site of metastatic disease was the right retropulmonary lymph nodes. With the goal of increasing local control and potentially causing an abscopal effect, she underwent radiotherapy (24 Gy in three fractions of 8 Gy). She then received an additional four cycles of pembrolizumab. Unfortunately, disease progression was discovered in the liver shortly thereafter. She received olaparib (400 mg twice per day) for 2 months but without response, and she died in October 2016.

## Discussion and conclusions

In the past few decades, overall survival has not improved for women with OCS; the median overall survival is less than 2 years. Currently, there is no clear evidence to establish consensus guidelines for systemic management of OCS [10].

Published data evaluating the benefit of chemotherapy are scarce, and treatment recommendations are generally based on a few nonrandomized prospective studies and some retrospective analyses. Adding to this difficulty is the common exclusion of patients with OCS from prospective therapeutic clinical trials. Active agents given to patients with OCS include carboplatin, cisplatin, ifosfamide, paclitaxel, doxorubicin, and dacarbazine [1, 10]. Common treatment combinations include platinum plus paclitaxel and platinum plus ifosfamide, although the benefit of multiagent chemotherapy over single-agent chemotherapy is unclear. The overall response rate (ORR) to platinum-based chemotherapy for patients with OCS varies between 25 and 70%, whereas median overall survival ranges from 8 to 16 months [11]. There is little evidence regarding the effectiveness of second-line therapies. In a study of single-agent ifosfamide, an ORR of 17.9% in patients with recurrent disease was recorded [12]. Owing to the high rate of recurrence, even for those with early-stage disease, adjuvant systemic therapy is generally considered, although there is no clear consensus on the standard first-line therapy in the adjuvant and metastatic setting [10].

Several studies have verified the role of molecular signaling pathways in the treatment of OCS. OCS most frequently contains high-grade serous components, which often contain a *TP53* mutation, and the precursor lesions may originate from normal-appearing fallopian tube epithelium that contains a *TP53* signature [13]. Given the poor response of OCS to standard available therapies, researchers have sought insight from molecular characterizations such as next-generation whole-exome sequencing [14]. Unsurprisingly, given the high rate of serous adenomatous components in OCS, the most common alteration is in *TP53* [13]. Other alterations described include mutations in *H2A* and *H2B*, deletions of *TP53* and *MBD3*, and amplification of chromosome segments containing *PIK3CA*, *TERT*, and *MYC*. However, few of these mutations are directly druggable [14].

Nevertheless, some EOCs have been known to induce a strong immune response characterized by high tumor T-cell infiltrates. As such, immune checkpoint inhibitors and combinations with chemotherapy, anti-angiogenesis agents, poly (ADP-ribose) polymerase (PARP) inhibitors, and other immune active compounds are under active investigation. Immunologic effector cells may be blocked by inhibitory regulatory pathways controlled by specific molecules often called immune checkpoints. These checkpoints serve to control or turn off the immune response when it is no longer needed to prevent tissue injury and autoimmunity [15]. Immune responses to ovarian cancer appear to vary by histologic subtype; high-grade serous cancers are most likely to be associated with a prognostically favorable tumor-infiltrating lymphocyte response. Classification of various histologic subtypes of ovarian cancers on the basis of tumor-infiltrating lymphocytes and PD-L1 expression revealed that type I patterns were more common in high-grade serous cancers and type IV patterns predominated in other histologic subtypes [16].

A phase Ib trial evaluated the safety and antitumor activity of pembrolizumab, an anti-PD-1 antibody, in patients with PD-L1-positive advanced solid tumors. One of the 26 patients with advanced EOC obtained a complete response and two patients experienced a partial response. The ORR was 11.5%, and the most common adverse events reported were fatigue, anemia, and decreased appetite [17]. Pembrolizumab has been studied in various scenarios in EOC: neoadjuvant setting, maintenance treatment, and recurrent or metastatic disease [18].

Another anti-PD-1 antibody, nivolumab, has also been studied in ovarian cancer. In a phase II trial, nivolumab was administered in patients with platinum-resistant EOC. Two complete responses were observed, as well as one partial response. The ORR was 15%, and the disease control rate was 45% [19]. Avelumab, a fully-humanized anti-PD-L1 IgG1 antibody, was studied in a phase I trial in patients with recurrent or refractory EOC. The ORR was 9.7% and the disease control rate was 54% [20]. A summary of the results of single-agent trials of PD-1/PD-L1 inhibitors is presented in Table 1. Ongoing studies are also evaluating the role of an anti-CTLA-4 antibody. Independent from the prognostic significance of PD-L1 expression, PD-L1/PD-1 receptor B7/CTLA-4 interactions are important immune escape mechanisms, allowing tumor progression [18].

**Table 1:** Clinical trial results for PD-1 and PD-L1 inhibitors in ovarian cancer

Result	Agent				
	Nivolumab [19]	Pembrolizumab [17]	Avelumab [20]	Atezolizumab [23]	Durvalumab [24]
No. of patients	20	26	124	12	15
Prior therapies	≥4 in 55% of cases	≥5 in 38.5% of cases	≥3 in 65.3% of cases	≥6 in 58% of cases	median 4
PD-L1+ prevalence	80% (IC 66%)	100% (> 1% TC)	77% (IC 66%)	83% (> 1% TC)	73% (> 5% TC)
Overall response rate	15%	11.5%	9.7%	25%	Not reported
Median progression-free survival	3.5 months	Not reached	2.6 months	2.9 months	Not reported
Median overall survival	20 months	Not reached	10.8 months	17.4 months	Not reported

*IC* immune cells, *TC* tumor cells

To the best of our knowledge, this case report represents the first data on the use of pembrolizumab in OCS. The objective response in our patient suggests that OCS, like EOC, is an immunogenic malignancy. Interestingly, our patient was heavily pretreated with multiple locoregional and systemic therapies. It is unknown whether radiotherapy may have contributed to the objective response according to the suggested abscopal effect [21]. The biologic phenomenon underlying this effect is not completely understood, but it may be mediated by immunologic mechanisms [22].

In conclusion, pembrolizumab in this patient appeared to provide some tumor control in multifocal metastatic sites, despite the effects being short-lived. OCS should be evaluated in prospective trials and further work is needed to identify patients most likely to respond to this type of intervention.

## Abbreviations

EOC: Epithelial ovarian cancer
OCS: Ovarian carcinosarcoma
ORR: Overall response rate
